# Supplementary material for: Deficient Incorporation of Rabies Virus Glycoprotein into Virions Enhances Virus-Induced Immune Evasion and Viral Pathogenicity
Source: Viruses. 2019 Mar 4;11(3):218. doi: 10.3390/v11030218 (PMC6466124; doi:10.3390/v11030218)
Supplement: Supplementary file 1 [file viruses-11-00218-s001.pdf]

Supplementary Materials

Supplementary Figures

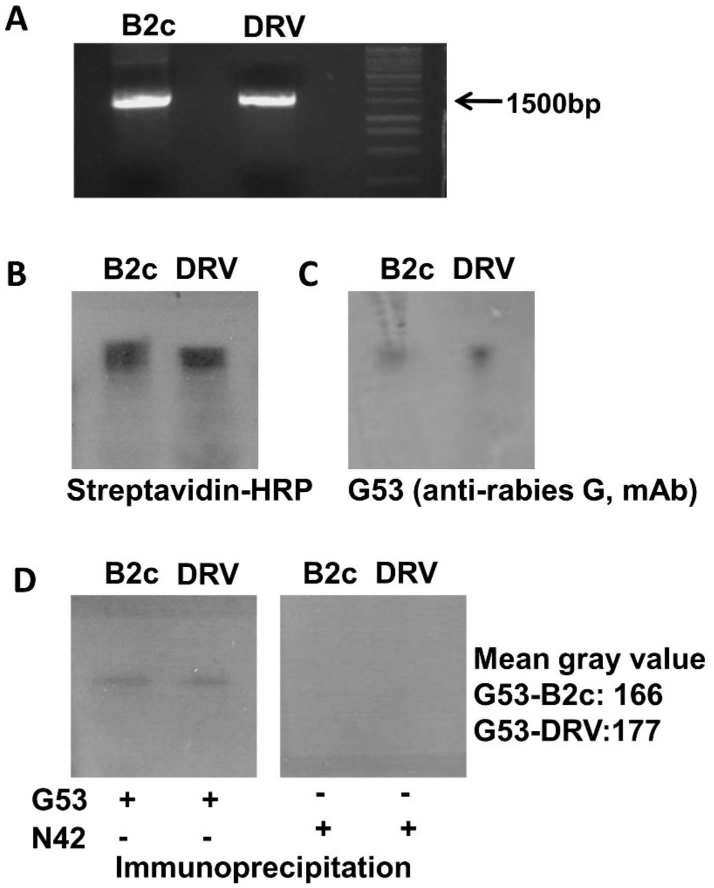

Figure S1. In vitro **translation of different RABV-G and their binding affinities to anti-G antibodies.** (A) PCR-generated fragments containing the T7 promoter and purified by GEL purification kit. (B) The same level of RABV-G proteins synthesized by T7 Quick Coupled Transcription/Translation System were added together with Transcend tRNA in a 50-μl reaction volume for 90 min at 30°C. RABV-G protein production was detected with G53 and Streptavidin-HRP by Western blot. (C) Immunoprecipitation was carried out at 4°C overnight using G53 and N42. The resulting immune complexes were bound to Protein A-agarose (Sigma) and analyzed by Western blot. Protein bands were visualized by chemiluminescent assay, and mean gray value (MGV) was analyzed by ImageJ.

Supplementary Methods

In vitro **translation, immunoprecipitation, and PAGE analysis of the G protein.** B2c G and DRV-G were used as templates to generate fragments containing the T7 promoter, which was added with methionine and biotinylated lysine-tRNA complex to the TnT Quick Master Mix and incubated in a 50-μl reaction volume for 90 min at 30°C (Promega). Immunoprecipitation was carried out at 4°C overnight using monoclonal antibody G53. The resulting immune complexes were bound to Protein

G-agarose (Sigma, United States) and analyzed by 10% SDS-PAGE. Following electrophoresis, the gel was transferred to PVDF membrane and probed with Streptavidin-AP (Promega). Protein bands were visualized by chemiluminescent assay and mean gray values (MGVs) were analyzed by ImageJ.
